# Serum microRNAs in male subfertility—biomarkers and a potential pathogenetic link to metabolic syndrome

**DOI:** 10.1007/s10815-017-0989-0

**Published:** 2017-06-29

**Authors:** Dorota Trzybulska, Johannes Bobjer, Aleksander Giwercman, Christos Tsatsanis

**Affiliations:** 1 Molecular Reproductive Medicine, Department of Translational Medicine, Lund University, CRC, Building 91, Plan 10, Jan Waldenströms gata 35, SE 20502 Malmö, Sweden; 2 Department of Urology, Skåne University Hospital Malmö, Jan Waldenströms gata 7, SE 20502 Malmö, Sweden; 3 Reproductive Medicine Centre, Skåne University Hospital Malmö, Plan 3, Jan Waldenströms gata 35, SE 20502 Malmö, Sweden; 4 Department of Clinical Chemistry, School of Medicine, University of Crete, Voutes, PO Box 2208, 71003 Heraklion, Crete Greece

**Keywords:** Serum microRNA, Male subfertility, Metabolic disturbances

## Abstract

**Purpose:**

The purpose of the study was to identify serum microRNAs providing a link between male subfertility and metabolic syndrome (MetS) and validate their diagnostic potential.

**Methods:**

Sera were analyzed for fertility and MetS-related parameters in subfertile men (*n* = 79) and controls (*n* = 38). Literature review identified miR-155-5p, miR-122-5p, miR-200a-3p, and miR-200c-3p which previously were associated with parameters of fertility as well as metabolic disorders. They were measured in the sera using an absolute quantitation method (qPCR). In order to investigate the value of miRNAs in predicting subfertility, receiver operating characteristic analysis was done.

**Results:**

Subfertile men had higher concentrations of miR-155-5p than controls (*p* = 0.003) and for miR-200c-3p, the difference was borderline statistically significant (*p* = 0.05). miR-155-5p and miR-200c-3p were also associated with subfertility in men with no metabolic disturbances (*p* = 0.008, *p* = 0.004, respectively). This association was abrogated if any component of MetS was present. The combination of miR-155-5p and miR-200c-3p with follicle-stimulating hormone, being a well-established subfertility parameter, resulted in an overall diagnostic power of AUC = 0.87, which was even higher when men without MetS components were analyzed (AUC = 0.93). Regarding MetS components, statistically significant correlations were found between miR-122-5p and fasting triglycerides, and waist circumference, but no association with subfertility was identified.

**Conclusions:**

Among the four miRNAs analyzed, none of them was associated both with male subfertility and MetS components. The ability of miR-155-5p and miR-200c-3p to identify subfertile men was partly overruled by the presence of metabolic disturbances.

**Electronic supplementary material:**

The online version of this article (doi:10.1007/s10815-017-0989-0) contains supplementary material, which is available to authorized users.

## Introduction

Infertility is a global health problem and it is estimated that it is experienced by one out of 6–7 couples in the Western world [1, 2]. A predominant or partial contribution of a male factor is supposed to occur in half of the cases, with only a minor proportion of those conditions having known causes [1–3]. An association between male subfertility and androgen deficiency has been reported and the latter seems to be linked to the risk of metabolic syndrome (MetS) defined as the presence of at least three of the following risk factors: abdominal obesity, low high-density lipoprotein (HDL) cholesterol, and elevated fasting glucose, triglycerides or blood pressure [2, 4]. It has been demonstrated that all individual symptoms of MetS can be associated with impairment of male fertility through mechanisms not yet fully understood [2]. Among metabolic disorders, overweight and obesity come irrecusably to the fore. Obese men suffer from different pathological and clinical conditions compromising spermatogenesis mainly due to the dysfunction of hypothalamic-pituitary-testis axis dysfunction which clearly links obesity to reduced fertility [5].

A number of lifestyle factors have been identified as affecting male fertility and, in many cases, contributing to epigenetic modifications which might have an impact on the development of MetS [6]. Among molecules acting pleiotropically in cellular functions, microRNAs (miRNAs) have been pointed out as potentially regulated by androgens [7] and interacting with multiple genes controlling spermatogenesis [1, 8] as well as metabolism [9]. MiRNAs belong to a class of small non-coding RNA molecules containing about 22 nucleotides. By targeting the 3′ untranslated region (3′-UTR) of mRNAs, each miRNA regulates mainly post-transcriptionally expression of multiple genes and thereby, their action is classified as one of the epigenetic mechanisms [10, 11]. The role of miRNAs in affecting male fertility is not yet completely known but since they are active players in inflammation [12, 13], they may represent a link between impairment of male fertility and MetS.

MiRNAs are also present in serum/plasma as cell-free circulating miRNAs and can be utilized as biomarkers communicating changes in tissues being affected by several diseases. In a study by Tsatsanis et al., miR-155-5p appeared as a new and reliable biomarker of male subfertility. However, in that study a control population with a population of subfertile men were compared without having any reference values for miR-155-5p concentration in serum [14]. Additional studies have pointed to other miRNAs in the testicular tissue, seminal plasma or spermatozoa that also might be associated with male subfertility [15–18]. Whether changes in those miRNAs are reflected in changes in their serum levels is not known.

In the present study, we performed literature review and identified four miRNAs which in previous reports were linked to metabolic disturbances as well as to male fertility. However, these studies were based on investigation of independent cohorts. Therefore, the aim of current study was to assess the levels of these miRNAs in a cohort of men, with well-characterized reproductive as well as MetS parameters, in order to elucidate whether these miRNAs represent a pathogenetic link between male subfertility and metabolic disturbances. The secondary aim was to elucidate whether measurement of serum miRNA is a better tool in prediction of male subfertility as compared to analysis of follicle-stimulating hormone (FSH) which is a well-established serum marker of spermatogenesis.

## Materials and methods

### Study groups

Seventy-nine subfertile men were randomly chosen from a cohort recruited in an earlier study [4] at the Reproductive Medicine Center, Lund University Hospital, Malmö, Sweden. Subfertility was defined as sperm concentration below 20 × 10^6^/mL in at least two independent semen samples in men whose partner had not conceived within one year. As controls, 38 men selected from the general population were chosen. These men had not received any fertility treatment prior or during recruitment. The workup encompassed medical history and extensive clinical as well as laboratory evaluation of fertility- and MetS-related parameters. MetS was defined according to the criteria of the National Cholesterol Education Program Adult Treatment Panel II 2002 (NCEP-ATP III) [19]. Demographic data, anthropometric measurements, biochemical characteristics, and hormone levels are presented in Supplemental Table 1. Medical history and current medication are listed in Supplemental Table 2. This study was approved by the Regional Ethical Review Board in Lund, Sweden (no LU 2012/310). All subjects were recruited in 2012 and participated with written informed consent. The study was performed in accordance with the Declaration of Helsinki.

### Hormone and blood chemistry assays

Blood was collected between 8 and 10 a.m. after an overnight fasting. Hormone and blood chemistry assays were performed at the Department of Clinical Chemistry, Malmö. Total testosterone levels were determined by a two-step competitive immunoassay with an electro-chemiluminescence immunoassay (ECLI), imprecision (CV%) 7% at 3.0 nM and 4% at 15 nM. Luteinizing hormone (LH) and sex hormone binding globulin (SHBG) concentrations were measured by using a one-step immunometric sandwich assay with ECLI. Free testosterone was calculated from total testosterone (TT), SHBG, and a fixed albumin level (43 g/L) [20]. Lipid (LDL, HDL, total cholesterol, and triglycerides) concentrations were determined using standard enzymatic methods. To assess fasting plasma glucose an automated hexokinase method was applied. Fasting insulin levels in serum were determined with an immunometric sandwich assay. Fasting insulin and fasting glucose were used to calculate homeostatic model assessment (HOMA) index according to equation (f-insulin × f-glucose/22.5). Glycated hemoglobin (HbA_1c_) was measured with the VARIANT TURBO Hemoglobin A_1c_ Kit 2.0 program using cation exchange and gradient elution. High sensitivity C-reactive protein (hs-CRP) was analyzed in a Beckman AU5400 analyzer using Beckman reagents.

### Selection of miRNAs

To select miRNAs, literature review was performed applying the following criteria for selection: previously reported presence in serum, association with abnormalities in semen in subfertile men, and metabolic-related functions in at least one PubMed indexed paper. According to these criteria, miR-122-5p, miR-200a-3p, miR-200c-3p, and miR-155-5p were selected for further analysis. Supplemental Table 3 includes information and characteristics of the selected miRNAs.

### Analysis of miRNAs expression by RT-qPCR

RNA was extracted from 200 μL serum by using miRCURY™ RNA Isolation Kit (Exiqon, Denmark) according to the manufacturer’s instruction. Because of the lack of reliable endogenous miRNA, which might serve as a reference, the fixed amount of external miRNA UniSp6 was added at the beginning of the RNA extraction procedure, mainly to effectively monitor potential RNA loss during extraction. Total RNA (4 μL) was reverse transcribed by using Universal cDNA Synthesis Kit II (Exiqon, Denmark). To determine expression levels of selected miRNAs, standard curves were generated (10-fold dilution series of for each of them by using an artificial oligo template with a starting dilution of 150 fM). Concentration of miRNAs ranged between femto- (fM) and attomolar (aM). Reactions were carried out in duplicates using a negative control sample and UniSp6 by using ExiLENT SYBR® Green master mix kit and the Stratagene Mx3000P instrument with MxPro™ software. The thermal cycling conditions were as follows: denaturation 95 °C 10 min followed by 45 cycles of 95 °C for 10 s and 60 °C 60 s and post-qPCR melting curve formation: 95 °C 1 min, 55 °C 30 s, 95 °C 30s. Intra- and inter-assay coefficient of variability was determined for studied miRNAs (<2.41 and <3.98%, respectively). Calculations were performed based on the method described by Kroh et al. [21].

### Data analysis

The group characteristics are expressed as medians (interquartile ranges, IQR). Missing data regarding metabolic parameters excluded subjects from the analysis (two controls and one subfertile man). In order to compare levels of the four miRNAs between subfertile men and controls, Mann-Whitney *U* test was used. Since selected miRNAs were reported to be linked to the metabolic state (Supplemental Table 3), this analysis was performed for all subjects as well as for the subgroups not having any sign of MetS. Subsequently, we applied Spearman rank correlation test to see a possible link between the miRNAs and the individual MetS components, and additionally, sperm concentration.

In order to investigate the value of miRNAs, as compared to FSH, in predicting subfertility, receiver operating characteristic (ROC) analysis was done with the web-tool easyROC (v. 1.3). A non-parametric method was used for curve fitting and DeLong [22] method for confidence interval and standard error (SE) estimation [22]. The Youden index and associated cut-off points were used to measure overall diagnostic effectiveness [23]. We compared the area under the curve (AUC) for FSH as well as for those miRNA for which we found statistically significantly different levels in subfertile men and controls. Finally, the AUC for combination of FSH and miRNAs was calculated by using predicted probabilities generated in binary logistic regression. Similar analysis was done for subjects without any sign of MetS.

The differences between the groups and correlations were considered statistically significant at *p* < 0.05. The statistical analysis was done by using IBM SPSS Statistics software (v. 23.0, Chicago, IL).

## Results

### miR-155-5p

In the group of subfertile men, serum levels of miR-155-5p were significantly higher than in controls. A similar difference was still observed after removing cases with at least one MetS component (Table 1). However, when subjects with azoospermia and Klinefelter syndrome were excluded from the analysis, the difference was less prominent (Supplemental Table 4). There were no statistically significant correlations between miR-155-5p expression and MetS parameters (Table 2). A model including miR-155-5p adjusted for dyslipidemia (HDL) did not confound the power of miR-155-5p in predicting subfertility in our study group (Supplemental Table 5).

**Table 1:** Serum miR-155-5p, miR-200c-3p, miR-122-5p, and miR-200a-3p concentrations in controls and subfertile men

miRNA	All subjects			No MetS components^a^			At least one MetS component^a^			No MetS component, Klinefelter syndrome, and azoospermia^a^		
	Controls *n* = 38	Subfertile *n* = 79	*p* ^b^	Controls *n* = 22	Subfertile *n* = 39	*p* ^b^	Controls *n* = 14	Subfertile *n* = 39	*p* ^b^	Controls *n* = 22	Subfertile *n* = 25	*p* ^b^
miR-155-5p (aM) miR-200c-3p (aM) miR-122-5p (fM) miR-200a-3p (aM)	74.4 (75.5) 59.1 (37.2) 10.6 (22.6) 12.1 (24.3)	118 (79.9) 66.6 (46.5) 12.5 (25.3) 14.6 (18.7)	0.003 0.05 0.22 0.97	74.4 (70.6) 46.3 (36.4) 11.7 (20.8) 12.0 (13.1)	117 (60.8) 78.3 (46.9) 10.2 (24.3) 12.8 (23.0)	0.008 0.004 0.35 0.59	80.1 (93.1) 68.4 (38.1) 13.8 (81.5) 14.6 (29.2)	120 (83.1) 61.6 (47.0) 13.1 (26.3) 14.6 (16.6)	0.21 0.90 0.89 0.49	74.4 (70.1) 46.3 (36.4) 11.7 (20.8) 12.0 (13.1)	97.4 (76.6) 65.2 (46.5) 17.0 (26.1) 12.8 (15.4)	0.11 0.03 0.19 0.86

Data is presented as medians (IQR)

^a^Missing data for two controls and one subfertile man

^b^Mann-Whitney *U* test

**Table 2:** Correlation of miR-155-5p, miR-200c-3p, miR-122-5p, and miR-200a-3p with selected MetS-related parameters. Analysis was performed by using Spearman’s rank test for all subjects included in the study

Parameter^a^	miR-155-5p	miR-200c-3p	miR-122-5p	miR-200a-3p
Systolic blood pressure	0.07 (0.44)^b^	0.04 (0.70)	0.14 (0.13)	−0.19 (0.05)
Diastolic blood pressure	−0.02 (0.82)	0.06 (0.52)	0.17 (0.08)	−0.06 (0.52)
Fasting glucose	0.02 (0.84)	0.06 (0.55)	0.04 (0.64)	−0.03 (0.77)
Fasting triglycerides	0.17 (0.07)	0.11 (0.25)	0.28 (0.002)	0.12 (0.26)
HDL	−0.10 (0.28)	−0.10 (0.27)	−0.17 (0.08)	−0.07 (0.49)
Waist circumference	−0.03 (0.76)	0.01 (0.90)	0.26 (0.01)	−0.08 (0.43)

^a^Data available for 115 subjects

^b^The results are presented as Spearman’s rho (*p*-value)

### miR-200c-3p

Among subfertile men, miR-200c-3p expression was borderline significantly higher than in controls (Table 1). Median levels were significantly higher in subfertile men when individuals with manifestations of MetS were excluded from the analysis. We did not observe any statistically significant correlations between miR-200c-3p levels and metabolic parameters (Table 2).

### miR-122-5p

The levels of miR-122-5p did not differ significantly between controls and subfertile men, either with or without MetS components included (Table 1). Correlation analysis showed statistically significant associations with fasting triglycerides and waist circumference (Table 2). Moreover, we found a statistically significant correlation between miR-122-5p and sperm concentration (Supplemental Table 6). When the results were referred to overweight criterion, we found that miR-122-5p levels were significantly higher in subfertile men with BMI <25 (Supplemental Table 7).

### miR-200a-3p

For miR-200a-3p, there was no statistically significant difference in serum levels measured in subfertile men and controls (Table 1). As in the case of miR-122-5p, exclusion of subjects with metabolic disturbances did not change the result. No statistically significant association of serum levels of miR-200a-3p with fertility status or any of the metabolic parameters was observed (Table 2).

### miRNAs as predictors of subfertility

**Fig. 1:**
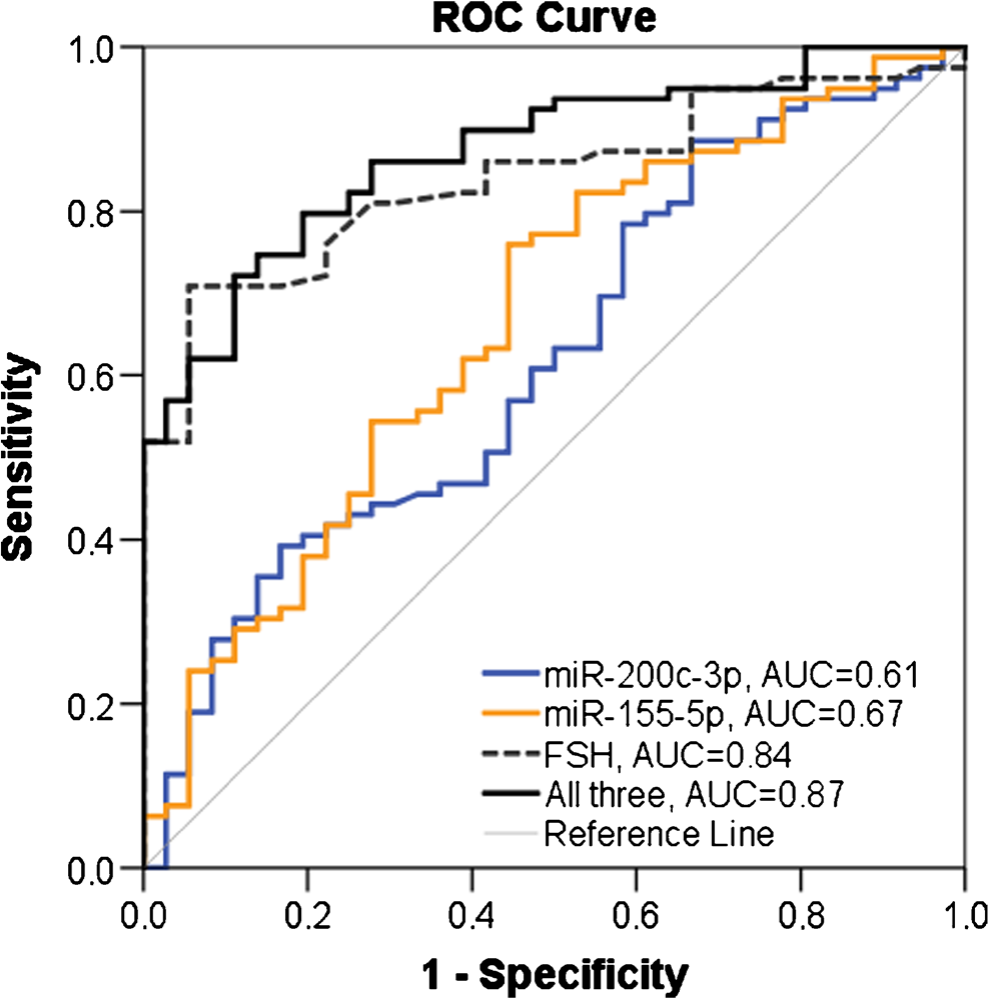
Receiver operating characteristic (ROC) curve plots for predicting fertility status based on miR-155-5p, miR-200c-3p, FSH, and when all three predictors were combined

For miR-155-5p a calculated cut-off level of 67.4 aM predicted subfertility with sensitivity 0.82 and specificity 0.47 (AUC = 0.67, *p* = 0.001). Corresponding values for miR-200c-3p were: cut-off level of 80 aM; sensitivity 0.39 and specificity 0.82 with AUC = 0.61, *p* = 0.001 (Table 3, Fig. 1). For FSH alone the cut-off value of 7.2 IU/L gave sensitivity of 0.71; specificity of 0.94 and AUC = 0.84 (*p* < 0.001).

**Table 3:** Receiver operating characteristics (ROC) metrics for miR-155-5p, miR-200c-3p, and FSH. The Youden index and associated optimal cut-off points were used to measure overall diagnostic effectiveness of discriminating between subfertile men and controls

	miR-155-5p (all subjects^a^)	miR-155-5p (no MetS components^b^)	miR-200c-3p (all subjects^a^)	miR-200c-3p (no MetS components^b^)	FSH (all subjects^c^)	FSH (no MetS components^b^)
Cut-off Sensitivity Specificity PPV NPV PLR NLR AUC	67.4 aM 0.82 (0.72–0.90) 0.47 (0.31–0.64) 0.77 (0.62–0.86) 0.56 (0.41–0.72) 1.56 (1.15–2.15) 0.37 (0.21–0.67) 0.67, *p* = 0.001	86.1 aM 0.78 (0.62–0.89) 0.59 (0.36–0.79) 0.78 (0.58–0.89) 0.59 (0.40–0.79) 1.89 (1.12–3.22) 0.38 (0.19–0.75) 0.71, *p* = 0.003	80.0 aM 0.39 (0.28–0.51) 0.82 (0.66–0.92) 0.82 (0.66–0.88) 0.39 (0.28–0.64) 2.13 (1.03–4.39) 0.75 (0.59–0.94) 0.61, *p* = 0.048	39.4 aM 0.95 (0.83–0.99) 0.41 (0.21–0.64) 0.75 (0.52–0.96) 0.82 (0.54–0.92) 1.61 (1.13–2.29) 0.12 (0.03–0.52) 0.72, *p* = 0.001	7.2 IU/L 0.71 (0.60–0.81) 0.94 (0.81–0.99) 0.97 (0.88–0.98) 0.60 (0.47–0.93) 12.8 (3.29–49.4) 0.31 (0.22–0.44) 0.84, *p* < 0.001	7.3 IU/L 0.78 (0.62–0.89) 0.96 (0.77–1.00) 0.97 (0.83–0.99) 0.70 (0.52–1.00) 17.1 (2.50–116.6) 0.24 (0.13–0.42) 0.90, *p* < 0.001

Data available for:

^a^79 subfertile men and 38 controls

^b^39 subfertile men and 22 controls

^c^79 subfertile men and 36 controls,

Results are presented as values (95% CI). For AUC *p*-value is given.

*PPV* positive predictive value, *NPV* negative predictive value, *PLR* positive likelihood ratio *NLR* negative likelihood ratio *AUC* area under the curve

The combination of miR-200c-3p and miR-155-5p with the established subfertility marker FSH slightly increased diagnostic power giving AUC = 0.87 (*p* < 0.001) (Fig. 1). In addition, if men with the presence of at least one component of MetS were excluded, the diagnostic power of the combination of all three parameters was further increased (AUC = 0.93, *p* < 0.001).

## Discussion

In the present study, we confirmed the previous finding regarding the association between levels of miR-155-5p and male subfertility [14]; however, none of the four miRNAs—miR-155-5p, miR-122-5p, miR-200a-3p, and miR-200c-3p—proved, when tested in same cohort, to be associated with both subfertility and parameters of MetS. MiR-200c-3p was also identified as an additional potential serum-based biomarker of male subfertility. Moreover, we demonstrated that the presence of metabolic disturbances at least partly masks the association of miR-200c-3p with subfertility. However, neither miR-155-5p nor miR-200-3p, when tested alone, emerged to be a better indicator of fertility status than FSH in our study group.

MiRNAs belonging to the miR-200 family have been associated independently with metabolism and inflammation [9] and their upregulation functionally related to male reproductive function when tested in seminal plasma [24]. Analysis in serum showed only a borderline statistically significant difference in miR-200c-3p levels between subfertile and control men. However, the association with subfertility was increased when subjects with at least one manifestation of MetS were excluded from the analysis, suggesting that low-grade systemic inflammation (LGSI) and metabolic disturbances may override its association with subfertility. Indeed, previous reports have shown that inflammatory factors such as TNFα and NF-κB, which are elevated in MetS-induced LGSI, potently induce expression of miR-200 family miRNAs [25], suppressing any fertility-related elevation of these miRNAs. Even though the miR-200c-3p had limited sensitivity and specificity as a predictor of subfertility, combination of miR-155-5p, miR-200c-3p, and the established marker of subfertility FSH resulted in higher AUC than any of the markers alone, suggesting that each of the markers is affected by independent factors, finally together affecting male subfertility.

Our findings confirm previous reports showing an association of miR-122-5p with parameters used to define MetS [26, 27] and highlights its potential to be used as a single serum marker that reflects multiple metabolic disturbances. The correlation found between miR-122-5p and sperm concentration in subfertile men might show an indirect link with metabolic disturbances.

The limitations of this study arise mainly from the number of samples analyzed, reducing the statistical power. Including men with more prominent features of MetS would potentially allow for identifying miRNAs showing clearer associations between MetS components and subfertility. An additional limitation is the fact that extracellular miRNAs expression does not always reflect intracellular changes [26].

In conclusion, this study demonstrated the potential of serum-derived miR-155-5p and miR-200c-3p as non-invasive biomarkers of subfertility. Our findings suggest that these miRNAs are associated with subfertility but this association is concealed in men with metabolic disturbances. Levels of these miRNAs in men with no metabolic disturbances may reflect pathophysiological changes that in the future could lead to MetS. Currently, functions of numerous miRNAs in relation to different pathomechanisms have been deciphered at the cellular and tissue levels. Nevertheless, still little is known if these changes in cell-free miRNAs can reflect both gonadal function and multifactorial disorders such as MetS. A follow-up longitudinal study will allow us to investigate if dynamics of changes in serum levels of miRNAs investigated in the present study in subfertile men without metabolic disturbances can predict the development of MetS.

## Electronic supplementary material

ESM 1 (DOCX 50 kb).
