# Supplementary material for: Serum microRNAs in male subfertility—biomarkers and a potential pathogenetic link to metabolic syndrome
Source: J Assist Reprod Genet. 2017 Jun 29;34(10):1277–82. doi: 10.1007/s10815-017-0989-0 (PMC5633576; doi:10.1007/s10815-017-0989-0)
Supplement: Supplementary file 1 — (DOCX 50 kb) [file 10815_2017_989_MOESM1_ESM.docx]

Supplementary material

**Serum microRNAs in male subfertility – biomarkers and a potential pathogenetic link to metabolic syndrome**

Journal of Assisted Reproduction and Genetics

**Table S1** Baseline characteristics of subfertile men and control group included to the study

**Table S2** Medical history and current medication in study groups

**Table S3** miRNAs related to male subfertility and metabolism selected by literature mining

**Table S4** miRNA levels in subjects without azoospermia and Klinefelter syndrome

**Table S5** Logistic regression of selected MetS parameters and miRNAs on accuracy to predict male subfertility. In models absence of selected parameters was set as a reference category.

**Table S6** Correlations between sperm concentration and miR-155-5p, miR-200c-3p, miR-122-5p and miR-200a-3p levels. The analysis was performed by using Spearman’s rank test for all subjects included in the study

**Table S7** miRNAs levels in subgroups of subjects divided according to BMI

**Supplemental Table 1** Baseline characteristics of subfertile men and control group included to the study

|  | **Controls (n=38)** | **Subfertile (n=79)** |
| --- | --- | --- |
| *Demographic data*  Age, (years)  Current smokers, n | 33.6 (15.2)  3 | 35.6 (7.4)  12 |
| *Anthropometric measurements*^a^  BMI (kg/m^2^)  Waist-hip ratio  Systolic blood pressure (mmHg)  Diastolic blood pressure (mmHg) | 25.0 (4.3)  0.87 (0.11)  131 (19)  78 (11) | 26.7 (4.4)  0.91 (0.07)  134 (15)  78 (10) |
| *Biochemical characteristics*^a^  HDL (mmol/L)  LDL (mmol/L)  Triglycerides (mmol/L)  Fasting insulin (mIU/L)  HbA_1c_ (mmol HbA_1c_/mol Hb)  Fasting glucose (mmol/L)  HOMA-IR (mmol/L x mIU/L)  Cholesterol (mmol/L)  LDL-HDL ratio  hs-CRP (mg/L)^b^ | 1.25 (0.40)  2.95 (1.55)  1.00 (0.75)  7.30 (6.10)  32.0 (5.00)  5.20 (0.70)  1.73 (1.00)  4.70 (1.50)  2.58 (2.13)  0.05 (0.09) | 1.30 (0.53)  2.9 (1.30)  1.00 (0.70)  8.70 (7.80)  33.0 (4.00)  5.20 (0.80)  1.90 (1.57)  4.80 (1.20)  2.23 (1.70)  0.13 (0.29) |
| *Hormone levels*^a^  FSH (IU/L)  cFT (nmol/L)  Total testosterone (nmol/L)  SHBG (nmol/L)  LH (IU/L) | 3.9 (2.6)  0.31 (0.12)  14.3 (7.43)  31.1 (20.5)  4.3 (2.4) | 8.1 (11.0)  0.29 (0.14)  13.8 (7.70)  30.0 (19.0)  6.2 (4.0) |
| The results are expressed as median (IQR)  ^a^ Data available for 36 controls  ^b^ Data available for 19 controls and 39 subfertile  Abbreviations: BMI, body mass index; HDL, high-density lipoprotein; LDL, low-density lipoprotein; HbA_1c_, glycated hemoglobin standardized to the International Federation of Clinical Chemistry and Laboratory Medicine method; HOMA-IR, homeostasis model assessment index of insulin resistance; PSA, prostate-specific antigen; hs-CRP, high-sensitivity C reactive protein; FSH, follicle-stimulating hormone; cFT; calculated free testosterone; SHBG, sex hormone-binding globulin; LH, luteinizing hormone | | |

**Supplemental Table 2** Medical history and current medication in study groups

|  | **Subfertile (n=79)** | **Controls (n=38)** |
| --- | --- | --- |
| ***Medical history, n***  Surgeries  Previous diseases  Current diseases  Azoospermia  Non-obstructive azoospermia  Hereditary diabetes type 2  Hereditary diabetes type 1  Orchiectomy  Varicocelectomy  Klinefelter syndrome  Previous testicular cancer  Previous malignancy  Previous orchitis  Previous epididymis  MetS (BP systolic criterion)  MetS (BP diastolic criterion)  MetS (BP criterion)  MetS (dyslipidemia criterion)  MetS (triglycerides criterion)  MetS (obesity criterion)  MetS (IR criterion)  MetS diagnose  No MetS components | 61  46  36  36  35  19  2  3  4  5  3  2  2  2  55  12  13  23  19  15  6  9  39 | 16  11  1  0  0  3  1  0  1  0  0  0  0  0  22  6  5  7  6  6  1  3  22 |
| ***Medication, n***  Painkillers (last 3 months)  Paracetamol (last 3 months)  Regular medication  Nonsteroidal anti-inflammatory drugs (last 3 months)  Acetylsalicylic acid (last 3 months)  Blood pressure decreasing treatment  Lipid decreasing treatment  Cortisone  Levaxine  Insulin treatment | 63  46  30  24  6  3  2  2  2  1 | 25  19  7  12  1  1  0  0  0  0 |

**Supplemental Table 3** miRNAs related to male subfertility and metabolism selected by literature review

| miRNA | **Association with reproduction** | **Association with metabolic disturbances** | **Model** | **Reference** |
| --- | --- | --- | --- | --- |
| miR-122-5p | Increased expression in asthenozoospermia and decreased in azoospermia |  | Human | <https://www.ncbi.nlm.nih.gov/pubmed/21933900> |
|  | Elevated expression in infertile males with semen abnormalities |  | Human | <https://www.ncbi.nlm.nih.gov/pubmed/23327642> |
|  | Predominately expressed in post-meiotic male germ cells, involved in chromatin remodeling during spermatogenesis |  | Mouse | <https://www.ncbi.nlm.nih.gov/pubmed/15901636> |
|  |  | Association with IR and regional adiposity in adults and IR in children | Human | <https://www.ncbi.nlm.nih.gov/pubmed/28183786> |
|  |  | Strong association with the risk of developing metabolic syndrome and type 2 diabetes in the general population | Human | <https://www.ncbi.nlm.nih.gov/pubmed/27899485> |
|  |  | Association with hepatic steatosis and fibrosis | Human | <https://www.ncbi.nlm.nih.gov/pubmed/24313922> |
|  |  | Elevated circulating miR-122-5p is associated with obesity and insulin resistance in young adults | Human | <https://www.ncbi.nlm.nih.gov/pubmed/25515554> |
|  |  | Involved in hepatic cholesterol and lipid metabolism | Mouse | <https://www.ncbi.nlm.nih.gov/pubmed/16258535> |
|  |  | Key regulator of cholesterol and fatty acid metabolism | Mouse | <https://www.ncbi.nlm.nih.gov/pubmed/16459310> |
|  |  | Up-regulated in white adipose tissue during the developments of obesity | Mouse | <https://www.ncbi.nlm.nih.gov/pubmed/22496873> |
|  |  | Decreased in circulation in patients after bariatric surgery in obese non-diabetic patients | Human | <https://www.ncbi.nlm.nih.gov/pubmed/28344345> |
| miR-155-5p | Elevated in azoospermia and asthenozoospermia |  | Human | <https://www.ncbi.nlm.nih.gov/pubmed/21933900> |
|  | Elevated in subfertile men |  | Human | <https://www.ncbi.nlm.nih.gov/pubmed/25740880> |
|  | One of key components of a complex developmental switch controlling Gnrh promoter activity, whose correct function is required for fertility |  | Mouse | https://www.ncbi.nlm.nih.gov/pubmed/27135215 |
|  |  | Involved in brown and beige fat cells development | Mouse | <https://www.ncbi.nlm.nih.gov/pubmed/23612310> |
|  |  | Deficiency leads to atherosclerosis, increased white adipose tissue obesity, and non-alcoholic fatty liver disease | Mouse | <https://www.ncbi.nlm.nih.gov/pubmed/27856635> |
|  |  | Overexpressed in the liver changes expression of genes associated with lipid metabolism | Mouse | <https://www.ncbi.nlm.nih.gov/pubmed/25799309> |
|  |  | miR-155-bearing adipocyte-derived microvesicles can mediate M1 macrophage polarization, which regulates insulin signalling and glucose uptake in adipocytes | Mouse | <https://www.ncbi.nlm.nih.gov/pubmed/27671445> |
|  |  | Regulates lipid metabolism in liver and its deregulation may lead to hepatic steatosis in patients with diabetes. | Mouse | <https://www.ncbi.nlm.nih.gov/pubmed/23991091> |
|  |  | Deficiency increases adipogenic, insulin sensitivity and limits inflammation in white adipose tissue. Targeting miR-155-5p may improve obesity resistance. | Mouse | <https://www.ncbi.nlm.nih.gov/pubmed/26953132> |
| miR-200a-3p | Elevated in azoospermia and asthenozoospermia |  | Human | <https://www.ncbi.nlm.nih.gov/pubmed/21933900> |
|  | One of key components of a complex developmental switch controlling Gnrh promoter activity, whose correct function is required for fertility |  | Mouse | https://www.ncbi.nlm.nih.gov/pubmed/27135215 |
|  |  | Overexpression of miR-200a in β-cells causes their massive apoptosis | Mouse | <https://www.ncbi.nlm.nih.gov/pubmed/25985365> |
|  |  | Decreased expression in obese patients with diabetes, glucose intolerance and normoglycemic as well as ob/ob mice | Human/Mouse | <https://www.ncbi.nlm.nih.gov/pubmed/24758184> |
|  |  | miR-200a-3p silencing increases the expression level of leptin receptor and insulin receptor substrate 2, reduces body weight gain, and restores liver insulin responsiveness. Thus, it could be a new target for the treatment of obesity | Mouse | <https://www.ncbi.nlm.nih.gov/pubmed/24394757> |
| miR-200c-3p | Elevated in azoospermia and asthenozoospermia |  | Human | <https://www.ncbi.nlm.nih.gov/pubmed/21933900> |
|  | One of key components of a complex developmental switch controlling Gnrh promoter activity, whose correct function is required for fertility |  | Mouse | https://www.ncbi.nlm.nih.gov/pubmed/27135215 |
|  |  | Deletion of miR-200 family miRNAs improves β-cell function | Mouse | <https://www.ncbi.nlm.nih.gov/pubmed/25985365> |
|  |  | Dysregulated in non-alcoholic fatty liver disease | Human/Rat | <https://www.ncbi.nlm.nih.gov/pubmed/25562147> |
|  |  | Down-regulated in white adipose tissue during the development of obesity | Mouse | <https://www.ncbi.nlm.nih.gov/pubmed/22496873> |

**Table S4** miRNA levels in subjects without azoospermia and Klinefelter syndrome

| miRNA | **Controls**  n=38 | **Subfertile**  n=43 | ***p*^a^** |
| --- | --- | --- | --- |
| miR-155-5p (aM)  miR-200c-3p (aM)  miR-122-5p (fM)  miR-200a-3p (aM) | 74.4 (75.5)  59.1 (37.2)  10.6 (22.6)  12.1 (24.3) | 103.1 (78.3)  57.4 (49.2)  15.0 (15.9)  14.6 (17.9) | 0.06  0.14  0.36  0.97 |
| Data is presented as medians (IQR)  ^a^Mann-Whitney *U* test | | | |

**Table S5** Logistic regression of selected MetS parameters and miRNAs on accuracy to predict male subfertility. In models absence of selected parameters was set as a reference category.

| **Variable** | **B** | | **SE** | **OR (95% CI)** | **-2 log-likelihood** | **Nagelkerke R^2^** | **Hosmer-Lemeshow *p*** | **Classification accuracy** |
| --- | --- | --- | --- | --- | --- | --- | --- | --- |
| **miR-122-5p** | -.006 |  | .005 | .994 (.985-1.003) | 144.8 | 3.3% | .163 | 70.1% |
| **miR-122-5p**  **obesity**  **obesity*miR-122-5p** | .026  2.92  -.071 | ^a^  * | .016  1.51  .034 | 1.027 (.995-1.060)  18.51 (.965-355.05)  .932 (.872-.995)* | 129.0 | 16.0% | .221 | 71.3% |
| **miR-122-5p**  **dyslipidemia (HDL)**  **dyslipidemia*miR-122-5p** | -.004  1.093  -.013 |  | .004  .658  .012 | .996 (.987-1.005)  2.982 (.821-10.84)  .987 (.964-1.010) | 136.5 | 7.7% | .082 | 71.3% |
| **miR-122-5p**  **dyslipidemia (triglycerides)**  **dyslipidemia*miR-122-5p** | -.005  .934  -.010 |  | .005  .716  .014 | .995 (.985-1.005)  2.544 (.625-10.35)  .990 (.964-1.016) | 137.7 | 6.2% | .382 | 71.3% |
| **miR-122-5p**  **blood pressure**  **blood pressure*miR-122-5p** | -.004  .985  -.024 |  | .004  1.003  .026 | .996 (.987-1.004)  2.677 (.375-19.12)  .976 (.928-1.027) | 137.9 | 6.1% | .504 | 70.4% |
| **miR-155-5p** | .010 | ** | .004 | 1.010 (1.003-1.018) | 138.6 | 10.3% | .281 | 70.1% |
| **miR-155-5p**  **dyslipidemia (HDL)**  **dyslipidemia*miR-155-5p** | .017  2.472  -.019 | **  *  * | .006  1.004  .008 | 1.017 (1.006-1.028)  11.85 (1.656-87.73)  .981 (.967-.996) | 128.3 | 16.8% | .784 | 71.3% |
| Abbreviations and labels: B, regression coefficient; SE, standard error; OR, odds ratio; CI, confidence interval; HDL, high density lipoprotein; * *p*<0.05; ** *p*<0.01; ^a^ borderline significance. | | | | | | | | |

**Table S6** Correlations between sperm concentration and miR-155-5p, miR-200c-3p, miR-122-5p and miR-200a-3p levels. The analysis was performed by using Spearman’s rank test for all subjects included in the study

|  | **miR-155-5p** | **miR-200c-3p** | **miR-122-5p** | **miR-200a-3p** |
| --- | --- | --- | --- | --- |
| **Sperm concentration** | -0.01 (0.93) | -0.30 (0.053) | **-0.36 (0.02)** | -0.09 (0.57) |
| Data available for 43 subjects  The results are presented as Spearman’s rho (*p*-value) | | | | |

**Table S7** miRNAs levels in subgroups of subjects divided according to BMI

|  | **miRNA** | **Controls^a^** | **Subfertile men^a^** | ***p*-value^b^** |
| --- | --- | --- | --- | --- |
| **BMI<25** | miR-122-5p (fM)  miR-155-5p (aM)  miR-200a-3p (aM)  miR-200c-3p (aM) | n=18  6.37 (20.9)  85.3 (65.3)  12.2 (24.8)  63.3 (37.1) | n=30  11.0 (21.3)  110.6 (63.5)  23.8 (29.6)  62.5 (38.7) | **0.03**  0.13  0.35  0.78 |
| **BMI≥25** | miR-122-5p (fM)  miR-155-5p (aM)  miR-200a-3p (aM)  miR-200c-3p (aM) | n=18  18.8 (46.5)  62.7 (86.8)  11.5 (14.5)  46.3 (36.1) | n=49  15.5 (27.8)  124.4 (78.9)  12.8 (15.8)  67.0 (53.3) | 0.34  **0.01**  0.59  0.07 |
| ^a^Data presented as medians (IQR)  ^b^Mann-Whitney *U* test | | | | |
